# Hemodynamic analysis of non-stenotic middle cerebral artery in patients with cerebral ischemia based on 4D flow MRI

**DOI:** 10.3389/fnins.2025.1502987

**Published:** 2025-07-10

**Authors:** Yue Chen, Jiali Sun, Ying Sui, Ying Shi, Jianxiu Lian, Ping Yang, Shuai Lin, Min Lyu, Na Li, Tong Zhang, Wei Wang

**Affiliations:** ^1^Department of MRI Room, The First Affiliated Hospital of Harbin Medical University, Harbin, China; ^2^Department of Advisory Clinical Scientist C&TS North, Philips Healthcare, Beijing, China; ^3^Department of Medical imaging, The Fourth Affiliated Hospital of Harbin Medical University, Harbin, China

**Keywords:** 4D flow MRI, cerebral ischemia, middle cerebral artery, hemodynamics, WSS = wall shear stress, peak velocity

## Abstract

**Objectives:**

Changes in cerebral hemodynamics in symptomatic states among patients with cerebral ischemia remain unclear. This study endeavors to investigate the characteristics of hemodynamics distribution within the non-stenotic middle cerebral artery (MCA) in patients with anterior circulation cerebral ischemia.

**Materials and methods:**

All subjects were prospectively recruited in this study. According to the clinical features of cerebral ischemia symptoms, they were divided into ischemia group and healthy group. MCA was further divided into proximal, curved, and distal segments based on the vascular morphology. Hemodynamic parameters, including flow, peak flow velocity, wall shear stress (WSS), pressure and energy loss (EL) were measured for each segment of the MCA in both groups using 4D flow MRI. The hemodynamic parameters of the proximal, curved, and distal MCA segments were compared within the group, and the influence of MCA morphology on hemodynamics was analyzed. Additionally, the hemodynamic parameters of the proximal, curved, and distal MCA segments were compared between the healthy and the ischemic groups to analyze the hemodynamic changes in the MCA among patients with cerebral ischemia.

**Results:**

A total of 50 MCAs were included in healthy group and 30 MCAs in ischemia group. There were no statistically significant differences in gender, age, heart rate, presence of hypertension and Body mass index (BMI) between the two groups. Results showed that the proximal segment had the highest blood volume (all *P* < 0.05), the M1 segment of MCA had the highest pressure (Max) and EL (Max and Avg) (all *P* < 0.01) in both groups. The peak blood flow velocity of the proximal and curved segments, the circumferential WSS of the distal segment, and the EL (Max and Avg) of the M1 segment in the ischemia group were lower than those in the healthy group (all *P* < 0.05).

**Conclusion:**

The distribution of hemodynamics in MCA is similar between anterior circulation cerebral ischemia patients and healthy group. In the ischemic group, the peak blood flow velocity, circumferential WSS, and EL of MCA were lower compared to the healthy group. The reduced flow velocity and the decrease of circumferential WSS in the curved section may be contributing factors to cerebrovascular events.

## Introduction

Over the past two decades (1990–2019), stroke has been the third leading cause of death worldwide (after ischemic heart disease and COVID-19). Ischemic stroke accounted for 65.3% of new strokes (Wu and Liu, 2023). In people under 70 years of age, the incidence and prevalence increased by 15 and 22%, respectively (Potter et al., 2022). Transient ischemic attack (TIA) is defined as a transient episode of neurologic dysfunction caused by focal brain ischemia without evidence of acute infarction on brain imaging and acute ischemic stroke (AIS) is defined as sudden neurologic dysfunction caused by focal brain ischemia lasting more than 24 h or with evidence of acute infarction on brain imaging, irrespective of symptom duration (Mendelson and Prabhakaran, 2021). Up to 11% of patients with untreated TIA may have an AIS within 7 days, rising to 18 within 90 days (Lee et al., 2021). This risk could be decreased by 80% if patients are immediately investigated and treated by stroke specialists (Lavallée and Amarenco, 2014). TIA serves as a major precursor to AIS and acts as an urgent warning sign. This underscores the critical importance of early identification and risk assessment of TIAs for short-term stroke risk (Foschi et al., 2022). At present, the diagnosis of this population mainly depends on the initial evaluation of the patient by clinicians (ABCD2 score, medical history, and physical examination), imaging examinations (MRI and CT confirm the presence of infarctions or other lesions in the brain, but early or mild lesions may not be detected or may result in false-negative results), and laboratory tests (Complete blood count, clotting time, blood sugar, may be affected by recent diet or other factors, resulting in inaccurate results) (Hankey, 2017; Scheitz et al., 2022; Bangad et al., 2023).

The vascular system maintains the homeostasis of cellular metabolism and physiological environment. Its dysfunction can lead to the occurrence and development of numerous dementia and neurological diseases (Morgan et al., 2021). Researchers illustrated that hemodynamic factors are associated with arterial disease damage (Liu and Zhang, 2012). In addition, the geometry of blood vessels (bifurcated, curved, and branched) can cause hemodynamic changes that would affect the development of vascular disease (Kim et al., 2015; Wong et al., 2020; Liu et al., 2022). Hemodynamic factors have been shown to play an important role in the formation and development of vascular diseases such as vascular dementia, atherosclerosis (AS), aneurysms (Sabayan et al., 2012; Michel, 2020). The M1 segment of the middle cerebral artery (MCA) extends from the end of the internal carotid artery to the first main partition of the MCA, where approximately one-quarter of cases of intracranial AS occur (Mazighi et al., 2006). The M1 segment with complex or high curvature may be a good site for AS due to changes in internal blood flow characteristics (Fan et al., 2008; Datir et al., 2011). Therefore, the hemodynamic characteristics of M1 segment have high research value.

4D flow MRI is a novel non-invasive technique for measuring hemodynamic parameters *in vivo*. It enables the acquisition of three-dimensional time-resolved volumes by encoding velocity in three spatial directions (Rizk, 2021). This technique allows for qualitative and quantitative assessment of flow velocity, flow, wall shear stress (WSS), pressure, and viscous energy loss (EL) throughout the cardiac cycle (Stankovic et al., 2014; Garcia et al., 2019). The information obtained from a single 4D flow MRI scan can be used both to study abnormal blood flow patterns at focal locations in the vasculature and to estimate whole-brain disturbances in the bloodstream. This local and global hemodynamic biomarker shows the potential to be sensitive to impending cerebrovascular disease and disease progression (Wåhlin et al., 2022). MRI has made a great contribution to the diagnosis of ischemic stroke (Potter et al., 2022). Diffusion-weighted imaging (DWI) is one of the most sensitive techniques to detect acute cerebral infarction, helping to quickly and accurately identify acute strokes. FLAIR images can better visualize white matter lesions, microhemorrhages, and edema, helping to distinguish between old and acute lesions. Combination of the two can improve detection rates of small vascular disease and other potential causes. SWI is useful in identifying occult vascular abnormalities or hemorrhagic spots. ASL is a non-invasive perfusion quantification technique that can assess cerebral blood flow (CBF) without a contrast agent, helping to identify areas of low perfusion and predict possible precursors of infarction. MRA is used to evaluate the condition of large blood vessels in and outside the cranial vessels, including aneurysms, dissections, stenosis, etc. However, none of these techniques can quantitatively and qualitatively analyze the hemodynamic parameters of a particular blood vessel. Previous studies have demonstrated the feasibility of 4D flow technology analyzing cardiovascular and cerebrovascular hemodynamics (Wen et al., 2019; Soulat et al., 2020; Wåhlin et al., 2022), with extensive researches focusing on macrovascular diseases of the heart (Isorni et al., 2020). The emergence of 4D flow is another technical supplement to the previous MRI technology in the field of hemodynamic research. Clinical practice has found that severe arterial stenosis and the reasons for cerebral ischemic events are often difficult to reverse. Due to the limitations of imaging technology, the detection of hemodynamics in the early stage of intracranial artery stenosis and its effect on cerebral ischemia are rarely studied. However, the hemodynamic mechanisms of non-stenotic major arteries in acute symptomatic states of ischemic cerebrovascular disease remains unclear.

Therefore, this study aimed to explore the hemodynamic pattern of MCA in healthy individuals by 4D flow MRI, and further analyzed the hemodynamic changes of non-stenotic MCA in the symptomatic state of patients with anterior circulation cerebral ischemia, so as to provide a basis for in-depth understanding of the pathogenesis in stroke.

## Materials and methods

This study has been approved by the Ethics Review Committee, and written informed consent has been obtained from the subjects.

### Study population

Patients with anterior circulation cerebral ischemia symptoms admitted to the Department of Neurology of the First Affiliated Hospital of Harbin Medical University from September 2022 to November 2023 and healthy people who came to the outpatient examination at the same time and patients with non-cerebral ischemia who came to the outpatient examination due to slight dizziness, headache, poor sleep and other reasons were prospectively recruited to identify as healthy volunteers, and all patients underwent MRI scans.

According to the presence or absence of cerebral ischemia symptoms, they were divided into two groups. In the ischemic group, there was an ischemic focus in the cerebral blood supply area of the anterior circulation (judged by DWI), or the patient has symptoms of anterior circulation cerebral ischemia, while the healthy group comprised individuals without symptoms of cerebral ischemia. Within 24 h of the MRI scan, the patient’s clinical information was collected, including body mass index (BMI), gender, age, heart rate, and hypertension. The inclusion criteria were as follows: (1) patients in the cerebral ischemia group who presented with at least one symptom of anterior circulation cerebral ischemia associated with MCA ischemia, such as monoplegia, hemiparesis, facial paralysis, aphasia with MRI scans performed within 3 days of symptoms onset; (2) patients in the healthy group who had never experienced the aforementioned symptoms of cerebral ischemia; (3) absence of intracranial artery stenosis as observed in MRA; (4) the MCA is C or S type; (5) patients in the ischemia group did not undergo medical or surgical treatment for ischemic symptoms before MRI scanning; (6) 18 years ≤ age ≤ 70 years. The exclusion criteria were as follows: (1) previous history of cerebral hemorrhage, brain tumor, epilepsy, vascular malformation, or cerebral infarction; (2) recent severe dizziness, vertigo, or headache attacks in the healthy group; (3) MCA with early bifurcation, repeat origin or aneurysm; (4) Presence of cardiogenic stroke; (5) contraindications to MRI examination; (6) poor image quality hindering analysis.

### Magnetic resonance protocol

A 3.0T MRI scanner (Ingenia Elition; Philips Healthcare, Best, the Netherlands) with a 32-channel head orthogonal coil was used. The scan sequence included the following: (1) three-dimension time-of-flight magnetic resonance angiography (3D-TOF-MRA): repetition time (TR)/echo time (TE) = 25 ms/3.5 ms; thickness = 1.4 mm; gap = −0.7 mm; field of view (FOV) = 194 mm × 194 mm × 84 mm; voxel = 0.7 × 0.7; Matrix = 275 × 275; (2) diffusion-weighted imaging (DWI): TR/TE = 2851 ms/47 ms; thickness = 4 mm; gap = 1 mm; FOV = 230 mm × 230 mm × 119 mm; voxel = 2.05 × 2.56; matrix = 112 × 90; slices = 24; *b* = 1000;(3) fluid-attenuated inversion recovery (FLAIR): TR/TE = 8000 ms/125 ms; thickness = 6 mm; gap = 1 mm; FOV = 230 mm × 230 mm × 125 mm; voxel = 0.9 × 0.9; matrix = 256 × 256; slices = 18; (4) 4D flow: volumetric, time-resolved phase-contrast method was employed for acquiring 4D flow MRI data. The scanning parameters were as follows: TR = 5.5 ms, TE = 2.5 ms, flip angle = 8°, FOV = 160 mm × 160 mm, voxel = 0.7 × 0.7, matrix = 192 × 193, thickness = 1 mm, slices = 30, acceleration factor = 3, in-plane resolution = 0.71 mm × 0.71 mm, Velocity encoding (VENC) was set at 100 cm/s to prevent aliasing artifacts. The total scan time of the 4D flow was approximately 8–12 min, depending on the heart rate of each subject. The finger pulse was used as the trigger to record acquisitions at the end of the diastole. It can ensure that the sequence of image acquisition is synchronized with the ECG cycle.

### Image analysis

In this study, a curved MCA in the M1 segment was included. Curvature is defined as two straight lines drawn through the midline of the MCA on the coronal and axial 3D-TOF-MRA images, one from the end of the intracranial segment of the internal carotid artery to the M1 segment maximum curvature point of the MCA (straight line M1M1’) and the other from the M1 segment bifurcation of the MCA to the M1 segment maximum curvature point of the MCA (straight line M2M2’), if the acute angle of intersection of these two lines on the coronal or axial MRA image is greater than 10°, the vessel is defined as a curved MCA vessel (Bai et al., 2022). The curved MCA was divided into two forms: C-type (When the projection of the M1 segment blood vessel on the axial, coronal or sagittal plane exhibits a curved shape similar to the letter “C”, it is classified as a C-shaped.) and S-type (When the projection of the M1 segment blood vessel on the axial, coronal or sagittal plane takes a hyperbolic shape similar to the letter “S”, it is classified as an S-shaped. This shape can be regarded as consisting of two connected C-shaped parts.) as previous study mentioned (Han et al., 2014; Yu et al., 2018), Figure 1. The raw data from the 4D Flow MRI sequence were imported into the dedicated software CVI42 (Version 5.14.1, Circle Cardiovascular Imaging, Calgary, Canada) for post-processing. Pre-processing is performed at first, including automatic offset correction, signal aliasing correction, flow direction correction, and dynamic preview of all directions of the image to identify and exclude poor-quality images. Then the target blood vessel parallel was divided to the centerline and trace along the blood vessel. The software can be used to automatically analyze the flow velocity, vector, trace, streamline, WSS, relative pressure difference between the two planes, EL and other parameters of the blood flow change of the target blood vessel, and then evaluate the entire blood flow state by observing the blood flow change in a cardiac cycle (Stankovic et al., 2014).

**FIGURE 1:**
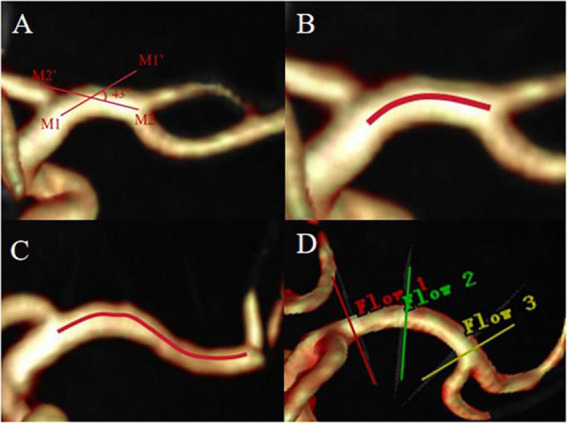
The acute angle between straight line M1M1’ and straight line M2M2’ is greater than 10°, defined as curved MCA **(A)**, C-type MCA **(B)**, S-type MCA **(C)**. **(D)** Shows the placement of slices, proximal (red line), curved (green line), distal (yellow line).

In the 4D flow image, the most curved position of the M1 segment of MCA was visually evaluated as the measurement cross-section for the curved segment. The junction between the carotid artery and MCA was used as the measurement plane for the proximal segment (1 mm after bifurcation), while the junction between the M1 segment and M2 segment of MCA was used as the measurement plane for the distal segment (1 mm before bifurcation) (Bai et al., 2022). The sections (in the proximal, curved, and distal segments) were placed perpendicular to the centerline in the analysis plane (Figure 1D) and blood volume, peak flow velocity, pressure (maximum, Max and average, Avg), axial WSS, circumferential WSS, 3D WSS (Max and Avg) and EL (Max and Avg) of the MCA M1 segment were measured during one cardiac cycle. Pressure is the difference between the cross-sections of the two vessels. The EL in this study is the total viscous EL. Viscous EL Viscous EL refers to energy dissipation caused by viscous friction inside the blood. EL parameters were reported as absolute values (mW) (Soulat et al., 2020). Two experienced radiologists independently conduct image analysis. The MCA on the ischemic side was analyzed in the ischemia group. Specifically, If there is a hyperintense ischemia foci on the exact MCA supply area on the DWI map, the MCA on the ipsilateral side of the lesion is analyzed. If there is no radiographic evidence of ischemia, the vessels to be analyzed were determined based on the clinical symptoms of the patient’s anterior circulation ischemia, then the MCA on the other side of the lesion is analyzed. Bilateral MCAs were analyzed in the healthy group.

**FIGURE 2:**
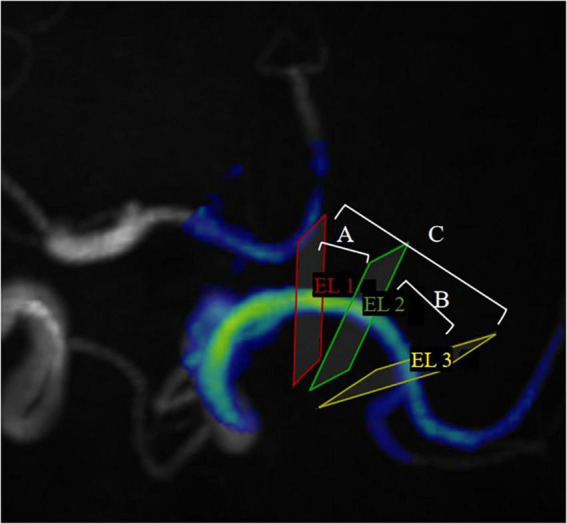
EL1, EL2 and EL3 represent proximal cross-sections, curved cross-sections, and distal cross-sections, respectively, in MCA. EL_A_ represents the energy loss between EL1 and EL2; EL_B_ represents the energy loss between EL2 and EL3; EL_C_ represents the energy loss between EL1 and EL3. To facilitate representation of statistics, we define EL_A_ as the EL in the proximal segment, EL_B_ as the EL in the curved segment, EL_C_ as the EL in the distal segment. (Pressure is expressed in the same way).

Hemodynamic parameters were measured for the proximal, curved, and distal segments including blood flow, peak velocity, axial WSS, circumferential WSS, 3D WSS (Max and Avg). Since the pressure and EL are the difference between two measurement planes, the Max pressure, Avg pressure, Max EL, and Avg EL of M1 segment of MCA from the proximal to the curved section, from the curved section to the distal section, and from the proximal to the distal section are measured separately. They were defined as proximal, curved, and distal segments to facilitate subsequent data presentation (Figure 2).

### Statistical analysis

Statistical analysis was performed using IBM SPSS (version 26.0, Chicago). Count data are expressed as percentages (*n*/%), and comparisons between groups were made using the chi-square (X2) test. The normality of continuous variables was tested using the Shapiro-Wilk test and the one-sample Kolmogorov-Smirnov test. The mean ± standard deviation is used for the normal distribution, and the median and interquartile ranges are used for the non-normal distribution. According to the symptoms of cerebral ischemia, it is divided into ischemic group and healthy group. According to the vascular morphology, MCA is divided into proximal, curved and distal segments. The hemodynamic parameters of the proximal, curved, and distal MCA segments were compared within the group (proximal_healthy_ VS curved_healthy_ VS distal_healthy_, proximal_ischemic_ VS curved_ischemic_ VS distal_ischemic_), and the influence of MCA morphology on hemodynamics was analyzed. Additionally, the hemodynamic parameters of the proximal, curved, and distal MCA segments were compared between the healthy and the ischemic groups (proximal_healthy_ VS proximal_ischemic_, curved_healthy_ VS curved_ischemic_, distal_healthy_ VS distal_ischemic_) to analyze the hemodynamic changes in the MCA among patients with cerebral ischemia. Comparisons between three groups (proximal, curved, and distal) were made using one-way ANOVA (*post hoc* analysis using LSD test) (normal distribution) or Kruskal-Wallis H test (non-normally distribution). Comparisons between the two groups (healthy and ischemic groups) were performed using either the Student’s *t*-test (normal distribution) or the Mann-Whitney U rank-sum test (non-normally distribution). A *P*-value less than 0.05 was considered statistically significant. Inter-reader consistency was assessed using the intraclass correlation coefficient (ICC). ICC ≤ 0.10 represents inconsistency; 0.10 < ICC ≤ 0.40 represents low consistency; 0.40 < ICC ≤ 0.60 represents general consistency; 0.60 < ICC ≤ 0.80 represents moderate consistency; ICC > 0.80 represents excellent consistency.

## Results

**FIGURE 3:**
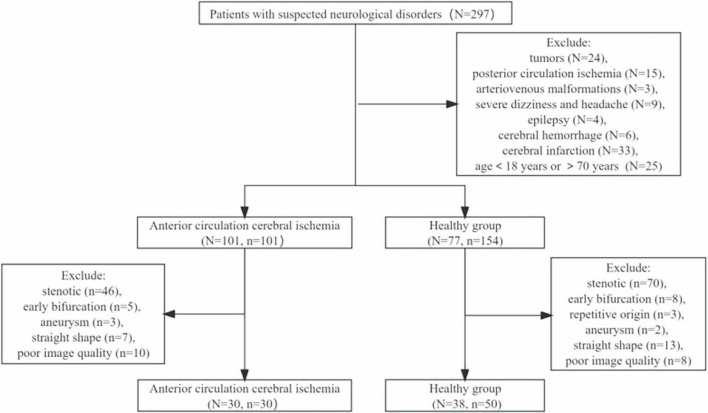
Flow chart of patient intake. N represents the number of patients, n represents the number of blood vessels.

**FIGURE 4:**
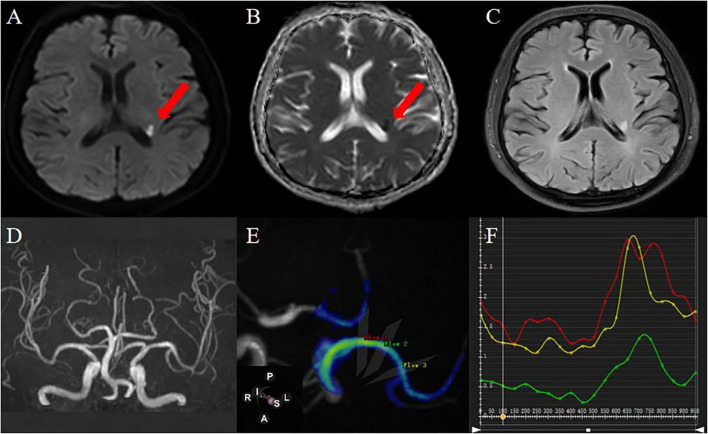
A 61-year-old male patient was admitted to the hospital because of weakness of the right limb. DWI **(A)** and ADC **(B)** images showed that the local diffusion near the posterior horn of the left ventricle was limited, suggesting acute cerebral infarction; FLAIR images **(C)** showed that there was no old cerebral infarction in the left hemisphere; 3D-TOF-MRA **(D)** shows that intracranial artery has no stenosis; 4D Flow image generates MCA simulation image, and the selected measurement flow position is as shown in the figure **(E)**:proximal (red), curved (green), distal (yellow) and blood flow parameter values automatically generated in each measurement plane are shown in **(F)** (the abscissa is the cardiac cycle/ms, the ordinate is the hemodynamic parameter, the same applies to other parameter measurements).

This study prospectively recruited 77 healthy individuals and 101 patients with symptoms of anterior circulation cerebral ischemia. Flow chart of patient intake was shown in Figure 3. 154 MCAs were analyzed in the health group, and ultimately 50 MCAs were enrolled. A total of 101 MCAs on the lesion side were identified and analyzed in the ischemic group, and 30 MCAs were ultimately included, the case was shown in Figure 4. In the healthy group, the average age was 55.36 ± 7.41 years old, and the heart rate was 73.50 (60.00, 83.00) times/min. In the ischemic group, the average age was 59.40 ± 6.77 years old, and the heart rate was 69 (62.75, 80.00) times/min. There was no significant difference in age, gender, heart rate, BMI, and presence of hypertension between the healthy group and the ischemic group (*P* > 0.05), as shown in Table 1.

**TABLE 1:** Clinical characteristics of patients in the healthy and ischemic groups.

Characteristic	Healthy group (*n* = 50)	Cerebral ischemia group (*n* = 30)	x^2^/t/Z	*P*
Male (*n*/%)	26/55.3	21/44.7	2.507^a^	0.113
Age (years)	55.36 ± 7.41	59.40 ± 6.77	−2.437^b^	0.314
Hypertension (*n*/%)	11/57.9	8/42.1	0.225^a^	0.635
BMI (kg/m^2^)	22.46 ± 2.35	23.40 ± 2.50	−1.703^b^	0.591
Heart rate (beats/min)	73.50 (60.00, 83.00)	69 (62.75, 80.00)	−0.165^c^	0.869

^a^*x*^2^ value;

^b^t value;

^c^Z value.

### Effect of MCA morphology on hemodynamics

**FIGURE 5:**
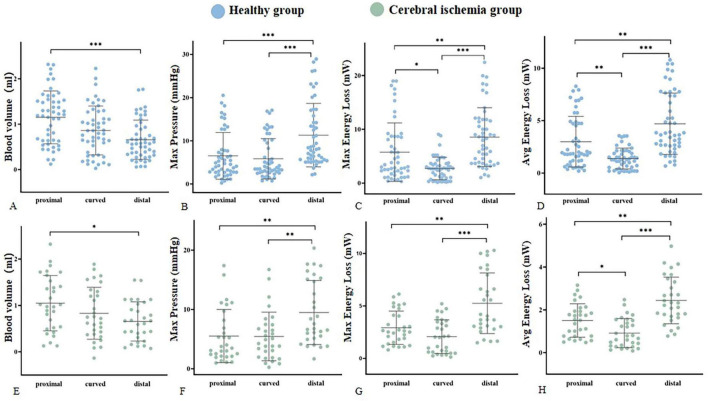
Hemodynamic parameters were statistically significantly different within groups. **(A–D)** Represent the blood volume, Max pressure, Max EL, and Avg EL of the healthy group (blue), respectively. **(E–H)** Represent the blood volume, Max pressure, Max EL, and Avg EL of the cerebral ischemia group (green), respectively. *<0.05, **<0.01, ***<0.001.

The hemodynamic distribution of the MCA in the cerebral ischemia group and the healthy group was similar (Figure 5). In both groups, the blood volume in the proximal segment of the MCA was the highest (*P* < 0.05), and decreased from proximal to distal (*P* < 0.05). The Max pressure, Max EL, and Avg EL in the near-to-far section (distal segment) were higher than those in the near-to-curved section (proximal segment) and the curved-to-far section (curved segment) (Max Pressure: *P* < 0.01, EL: *P* < 0.05). There were no statistically significant differences in peak blood flow velocity, axial WSS, circumferential WSS, Max 3D WSS, Avg 3D WSS, and Avg pressure between proximal, curved, and distal segments (Table 2).

**TABLE 2:** Intra-group comparison of hemodynamic parameters in different segments of MCA between healthy and ischemic groups.

Parameters	Healthy group (*n* = 50)				Cerebral ischemia group (*n* = 30)			
	Proximal	Curved	Distal	*P*1	Proximal	Curved	Distal	*P*2
Blood volume (ml)	1.14 ± 0.57	0.88 ± 0.60	0.58 (0.32, 0.94)	0.000^&^	1.05 ± 0.60	0.83 ± 0.56	0.66 ± 0.42	0.037^&^
Peak velocity (cm/s)	76.13 (70.14, 86.14)	76.22 ± 13.66	70.00 (63.32, 89.73)	0.380	70.80 ± 13.87	67.68 ± 20.32	68.97 ± 20.50	0.694
Axial WSS (pa)	0.56 (0.42, 0.82)	0.66 ± 0.38	0.60 ± 0.32	0.863	0.49 (0.31, 0.70)	0.60 (0.34, 0.77)	0.43 (0.29, 0.75)	0.680
Cir WSS (pa)	0.54 (0.38, 0.82)	0.55 ± 0.21	0.59 (0.44, 0.67)	0.419	0.50 ± 0.21	0.43 ± 0.17	0.48 ± 0.15	0.443
Max 3D WSS (pa)	1.01 ± 0.31	1.11 ± 0.28	1.03 (0.79, 1.29)	0.158	0.91 (0.79, 1.18)	1.05 ± 0.34	1.07 ± 0.44	0.846
Avg 3D WSS (pa)	0.70 (0.59, 0.86)	0.78 ± 0.19	0.73 ± 0.25	0.273	0.64 (0.55, 0.79)	0.70 ± 0.22	0.70 ± 0.29	0.873
Max pressure (mmHg)	4.66 (2.73, 7.83)	3.65 (2.65, 9.70)	8.32 (5.59, 16.12)	0.000^*#&^	3.62 (2.13, 8.57)	4.59 (1.94, 7.44)	7.17 (5.10, 15.14)	0.001^#&^
Avg pressure (mmHg)	0.10 (−1.03, 0.91)	−0.05 (−0.97, 1.02)	−0.64 ± 1.48	0.964	−0.08 ± 1.79	−0.23 ± 1.90	−0.15 ± 3.22	0.888
Max E L (mW)	3.25 (1.87, 8.69)	2.73 ± 2.09	8.14 (3.69, 11.84)	0.000^*#&^	2.58 (1.49, 4.28)	2.03 (0.58, 3.52)	4.12 (3.00, 8.10)	0.000^*#&^
Avg E L (mW)	1.94 (1.14, 4.78)	1.29 (0.52, 2.09)	3.73 (2.58, 7.45)	0.000^*#&^	1.54 (0.78, 1.98)	0.73 (0.31, 1.52)	2.28 (1.69, 3.51)	0.000^*#&^

*P*1 represents the difference between proximal segment, curved segment and distal segment in healthy people. *P*2 represents the difference between proximal segment, curved segment and distal segment in ischemia patients.

*Represents the difference between the proximal segment and curved segment.

^#^Represents the difference between the curved segment and distal segment.

^&^Represents the difference between the proximal segment and distal segment. Cir = Circumferential.

### Comparison of MCA hemodynamic parameters between healthy subjects and patients with anterior circulation cerebral ischemia

**FIGURE 6:**
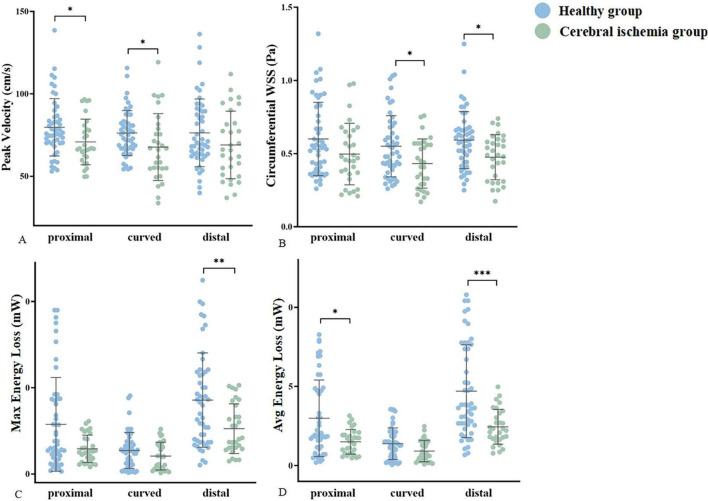
Statistically significant differences in hemodynamic parameters were compared between groups. **(A–D)** Represent the peak velocity, circumferential WSS, Max EL, and Avg energy loss of the healthy group (blue) and cerebral ischemia group (green), respectively. * < 0.05, ** < 0.01, *** < 0.001.

The peak blood flow velocity in the proximal and curved segments of the MCA was lower in the cerebral ischemia group than that in the healthy group (proximal: 70.8 ± 13.87 VS 76.13 (70.14, 86.14) *p* = 0.015, curved: 67.68 ± 20.32 VS 76.22 ± 13.66 *p* = 0.025). However, there was no statistically significant difference in the peak blood flow velocity between the two groups in the distal segment. The circumferential WSS in the curved and distal segments of the MCA was lower in the cerebral ischemia group when compared to the healthy group (curved: 0.43 ± 0.17VS0.55 ± 0.21 *p* = 0.019, distal: 0.48 ± 0.15 VS 0.59 (0.44, 0.67) *p* = 0.010), and there was no statistically difference in circumferential WSS between the two groups in the proximal segment. In the entire M1 section of the MCA, Max and Avg EL in the cerebral ischemia group were lower than those in the healthy group (Max:4.12 (3.00, 8.10) VS 8.14 (3.69, 11.84) *p* = 0.012, Avg: 2.28 (1.69, 3.51) VS 3.73 (2.58, 7.45), *p* = 0.001) (Figure 6 and Table 3).

**TABLE 3:** Hemodynamic comparison of different segments of MCA between healthy group and Cerebral ischemia group.

Position		Blood volume (ml)	Peak velocity (cm/s)	Axial WSS (pa)	Cir WSS (pa)	Max 3D WSS (pa)	Avg 3D WSS (pa)	Max pressure (mmHg)	Avg pressure (mmHg)	Max E L (mW)	Avg E L (mW)
Proximal	t/Z	0.679^a^	−2.435^b^	−1.416^b^	−1.551^b^	−0.55^a^	−1.357^b^	−0.895^b^	−0.378^b^	−1.729^b^	−2.351^b^
	P	0.750	0.015	0.157	0.121	0.956	0.175	0.371	0.706	0.128	0.028
Curved	t/Z	0.231^a^	2.042^a^	−0.497^b^	−2.336^a^	0.869^a^	1.741^a^	−0.109^b^	−0.035^b^	−1.004^b^	−1.804^b^
	P	0.804	0.025	0.619	0.019	0.121	0.440	0.913	0.972	0.315	0.071
Distal	t/Z	−0.099^b^	−1.471^b^	−0.900^b^	−2.575^b^	0.077^b^	0.508^a^	−0.87^b^	−0.735^a^	−2.708^b^	−3.498^b^
	P	0.921	0.141	0.368	0.010	0.722	0.253	0.385	0.462	0.012	0.001

Cir = Circumferential;

^a^t value;

^b^Z value.

### Inter-observer consistency analysis

20 cases were randomly selected for inter-readers consistency analysis of hemodynamic measurement parameters. There was good agreement between Inter-observer consistency analysis (ICC: 0.70–0.86) (Table 4).

**TABLE 4:** Inter-observer consistency analysis.

Position		Blood volume	Peak velocity	Axial WSS	Cir WSS	Max 3D WSS	Avg 3D WSS	Max pressure	Avg pressure	Max E L	Avg E L
Proximal	ICC	0.72	0.70	0.82	0.78	0.86	0.75	0.72	0.78	0.80	0.77
	P	0.000	0.000	0.000	0.000	0.000	0.000	0.000	0.000	0.000	0.000
Curved	ICC	0.85	0.77	0.74	0.72	0.85	0.75	0.73	0.75	0.79	0.71
	P	0.000	0.000	0.000	0.000	0.000	0.000	0.000	0.000	0.000	0.000
Distal	ICC	0.78	0.74	0.70	0.72	0.74	0.81	0.75	0.74	0.82	0.76
	P	0.000	0.000	0.000	0.000	0.000	0.000	0.000	0.000	0.000	0.000

ICC ≤ 0.10 represents inconsistency; 0.10 < ICC ≤ 0.40 represents low consistency; 0.40 < ICC ≤ 0.60 represents general consistency; 0.60 < ICC ≤ 0.80 represents moderate consistency; ICC > 0.80 represents excellent consistency. Cir = Circumferential.

## Discussion

4D flow MRI was used to measure the hemodynamic parameters of the curved MCA segments (proximal, curved, and distal) in the healthy and ischemic groups, and conducted comparative analysis within and between groups to investigate the relationship between intracranial artery vascular morphology and cerebral ischemia symptoms and hemodynamic changes. The results showed that the vascular morphology changed hemodynamics in a regular manner, with both groups exhibiting the highest proximal blood volume, and the Max pressure, energy losses (Max and Avg) throughout the M1 segment of MCA exceeded those of a single segment. The peak blood flow velocity, circumferential WSS, EL (Max and Avg) in the anterior cerebral ischemia group were lower than those in the healthy group.

### Effect of MCA morphology on hemodynamics

The results suggest that vascular geometry alters hemodynamics with regularity. The hemodynamic patterns of local anatomical positions in the patient group are similar to those in the healthy group. For evaluating whether different vascular geometries could cause hemodynamic changes, non-stenotic arteries were collected to avoid the influence of different degrees of lumen stenosis on hemodynamics, and arteries with similar curved morphology were enrolled. Specifically, in both groups, the greatest blood volume achieved in the proximal segment, possibly due to a larger luminal cross-sectional area (Hill et al., 2018; Nishi and Yasukawa, 2022). The brain lacks energy reserves and relies on a constant blood supply to maintain structural integrity and normal function (Morgan et al., 2021). This may mean that greater nerve damage will occur in the future when plaques occur in the proximal segment, compared to lesions occur in the curved and distal sections. A study supported this idea that in the M1 segment of MCA, patients with proximal stenosis score higher on the National Institutes of Health Stroke Scale at admission compared to patients with distal stenosis (Cho et al., 2009).

The Max pressure, Max EL, and Avg EL within the entire M1 segment of MCA, tended to surpass those of its individual segments, which could be attributed to the distance factor. In addition to the conversion between pressure, gravity, and kinetic energy, mechanical energy can also be converted into heat through friction between moving blood and stationary blood vessel walls. This heat can no longer be converted into mechanical energy, so energy is considered lost (Elbaz et al., 2017). According to the Pugachev Sveshnikov equation, we know that the total distance traveled is directly proportional to the energy consumed by blood sliding, and as it turns out there is a certain natural conservation law for it (Berezin and Zayats, 2018). Compared to the near to curved section and the curved section to far section, the EL of the entire M1 section (near to far section) is greater. This may mean that when the M1 segment is longer, EL will be greater, requiring higher compensatory ability from the body. Therefore, in clinical practice, more attention should be paid to the morphological characteristics of blood vessels, especially the M1 segment, in order to provide more diagnostic and therapeutic ideas for clinical practice.

### Hemodynamic changes in MCA in patients with cerebral ischemia

This study found that the peak blood flow velocity, circumferential WSS, and EL in patients with anterior circulation cerebral ischemia were lower than those in healthy individuals. Meanwhile, the curved segment in the cerebral ischemia group had lowest circumferential WSS and peak flow velocity. These findings showed despite the normal appearance of the blood vessels in the cerebral ischemia group, the microstructure may have been altered, resulting in stiffness or positive remodeling of the vessel wall, which led to a decrease in blood flow velocity (Liu et al., 2016).

Wall shear stress is the frictional force created by blood flow with the walls of blood vessels. It acts as a vector on the vessel wall in both the axial (direction of blood flow) and circumferential directions. The 3D WSS reflects the total WSS along the plane tangent to the local vessel surface (Frydrychowicz et al., 2009). In this study, we analyzed the axial and circumferential directions, as well as the 3D WSS, which was crucial for a comprehensive evaluation. Previous studies have demonstrated that WSS can impact the function of vascular endothelial cells and smooth muscle cells (Woo et al., 2023). Physiological WSS regulates endothelial function. Physiological laminar flow WSS promotes cell extension and orientation in the flow direction, inhibits proliferation, stimulates anti-inflammatory gene expression, and inhibits the expression of inflammatory pathways. WSS below or above its physiological value can induce changes in endothelial cell arrangement, polarization, and gene expression, and activate inflammatory response and remodeling processes (Roux et al., 2020). Low wall shear force and low flow rate are the primary causes of AS, particularly in vascular curvature (Soulis et al., 2014; Razavi et al., 2018). The circumferential shear and flow rate of the curved segment in the cerebral ischemia group are the lowest, which may align with the previous explanation. The findings of this study may shed light on the underlying hemodynamic mechanism of atherosclerotic cerebrovascular disease formation and development, with circumferential WSS potentially playing a dominant role in the occurrence and progression of stroke.

Energy loss is primarily attributed to the conversion of kinetic energy into heat energy through friction driven by viscosity, which is unavoidable (Kamphuis et al., 2019). Viscous EL was proportional to the flow rate (Akins et al., 2008). The cerebral ischemia group with a low flow rate exhibited a smaller EL, and our observation results are consistent with previous research. Energy loss is a parameter of cardiac workload caused by valve dysfunction or myocardial disease, applicable to the Fontan cycle (Itatani et al., 2017). The changes in EL are stimulated by the complex interaction between blood flow and intracardiac deformation, and the long-term existence of disproportionate EL may ultimately lead to increased myocardial load and ultimately lead to circulatory failure (Kamphuis et al., 2019). It may be of great significance in predicting ventricular load for various heart diseases, including valvular heart disease, cardiomyopathy, and congenital heart disease (Itatani et al., 2017). In cerebrovascular diseases, energy damage caused by the morphology of intracranial arteries may be one of the mechanisms leading to cryptogenic stroke, which may provide direction for clinical diagnosis and treatment.

### Limitations

This study has several limitations. First, we conducted a small sample study that requires prospective large-scale follow-up studies to confirm the results of this experiment. Second, the effect of upstream artery stenosis (extracranial carotid arteries, such as the bifurcation of internal and external carotid arteries) on the MCA is unknown, and the blood flow of the MCA in the ischemia group was abnormal, which may be caused by ipsilateral upstream carotid artery stenosis, and further research on the effect of upstream artery stenosis on it is still needed in the future. Third, our study revealed altered hemodynamics in the large arteries in the brain of patients with symptomatic cerebral ischemia, despite its normal appearance. However, intracranial arteries are difficult to obtain, and it may be of some significance to explore the mechanism of hemodynamic change in combination with high-resolution magnetic resonance imaging of vascular walls in the future.

## Conclusion

The hemodynamic distribution of MCA was found to be similar between the healthy group and the cerebral ischemia group. In the group with cerebral ischemia, the hemodynamics of MCA were lower than that of healthy group in peak blood flow velocity, circumferential WSS and EL. The reduced flow velocity and the decrease of circumferential WSS in the curved section may be contributing factors to cerebrovascular events. The results of this study may be helpful in revealing the hemodynamic rules of MCA in healthy individuals and patients with cerebral ischemia in the anterior circulation, and may offer hemodynamic mechanism of stroke from the perspectives of physiology and pathology.

## Data Availability

The raw data supporting the conclusions of this article will be made available by the authors, without undue reservation.
